# Low-loss, geometry-invariant optical waveguides with near-zero-index materials

**DOI:** 10.1515/nanoph-2022-0445

**Published:** 2022-11-07

**Authors:** Danqing Wang, Kaichen Dong, Jingang Li, Costas Grigoropoulos, Jie Yao, Jin Hong, Junqiao Wu

**Affiliations:** Department of Materials Science and Engineering, University of California, Berkeley, Berkeley, CA, 94720, USA; Miller Institute, University of California, Berkeley, Berkeley, CA, 94720, USA; Department of Mechanical Engineering, University of California, Berkeley, Berkeley, CA, 94720, USA; Lightgration LLC , Saratoga, CA, 95070, USA

**Keywords:** crosstalk, diffraction limit, geometry invariance, light scattering, optical waveguides, zero index

## Abstract

Optical materials with nearly zero refractive indices have driven emerging applications ranging from geometry-invariant optical tunneling, nonlinear optics, optical cloaking to thermal emission manipulation. In conventional dielectric photonic circuits, light scattering and back reflection at the waveguide bends and crossings leads to significant optical loss. Here we propose to use near-zero-index materials as a cladding layer for low-loss optical waveguides, where optical modes are tightly confined within the dielectric core region. Compared to conventional waveguides, the near-zero-index waveguides are superior in maintaining a high mode-filling factor for small device sizes close to the diffraction limit and reducing the crosstalk in between at a sub-wavelength separation. In addition, we found that light propagation is robust to waveguide bends in a small radius (∼µm) and geometry variation in the cross section. Hollow waveguides with near-zero-index cladding layers further support low-loss light propagation because materials absorption is minimized from the air core. Our work offers critical insights into future designs of low-loss and miniaturized photonic devices.

## 1 Introduction

Compared to electronic circuits, photonic chips that communicate using light offer a solution to faster computing with the potential for larger bandwidth, higher modulation speed, and lower power consumption [1, 2]. The programmable and scalable nature of photonic circuits may drive disruptive advances for future quantum communications [3, 4]. Current state-of-art, Si-based photonic chips, however, are limited to orders of magnitude lower device density than their electronic counterparts [5, 6]. Miniaturization of optical device elements is essential in increasing the integration density for future photonic circuits, but small-scale optical waveguides based on traditional dielectric materials exhibit pronounced radiative loss to the free space as well as increased crosstalk in between when the device size approaches the diffraction limit [7–9]. In addition, light scattering and back reflection at the bends and crossings of curved waveguides in Si photonics leads to significant optical losses [10, 11]. Ultra-low loss optical waveguides with a small device footprint are in need for future photonic circuits and hybrid-integrated quantum systems.

Epsilon-near-zero (ENZ) or near-zero-index materials emerge as a new class of materials that shows exotic optical behavior at their interface with conventional dielectrics [12, 13]. Such materials are typically achieved with transparent semiconducting oxides [14, 15], polaritonic materials [16], or structured photonic materials [17]. When the permittivity is close to zero, the wavelength as well as the phase velocity of light approach infinity within the ENZ material, which results in distinct optical responses such as geometry-invariant tunneling, optical cloaking, enhanced nonlinear optics, and directional thermal emission [15–20]. The light propagation is robust to geometry variation at the boundary, and the electromagnetic wave is highly confined at subwavelength scales within the ENZ materials [21–23]. Optical waveguides with an effectively zero permittivity of the bulk structure have been achieved at the cutoff frequencies of core–shell waveguides and Dirac cones of photonic crystals [24, 25]. Distinct from commercial optical fibers [26, 27], using ENZ materials as the cladding layer in waveguides would induce a much larger index contrast at the interface. Light propagation guided by ENZ materials could offer critical solutions to the significant optical losses in miniaturized photonic waveguides, a topic that has not been investigated.

Here we show that ENZ materials can serve as the cladding layer for low-loss and geometry-invariant optical waveguides, where electromagnetic modes are tightly confined within the dielectric core region. Unlike conventional dielectrics, ENZ-based waveguides show the advantages of maintaining a high mode-filling factor, especially for devices with sizes close to the diffraction limit. Such tight mode confinement is also tolerant to small permittivity variation in the cladding layer. Because of the greater numerical aperture of ENZ-based waveguides associated with a wider range of incident angles, we found that light propagation is robust to waveguide bends in a submicron radius and geometry variation in the cross section. The crosstalk between ENZ-based waveguides is also reduced compared to the dielectric ones, especially at a sub-wavelength separation. We further investigated air-core waveguides that support low-loss light propagation, where the materials absorption is minimized because of the air core. Our work offers prospects for future designs of low-loss and miniaturized photonic devices.

## 2 Results and discussion

**Figure 1:**
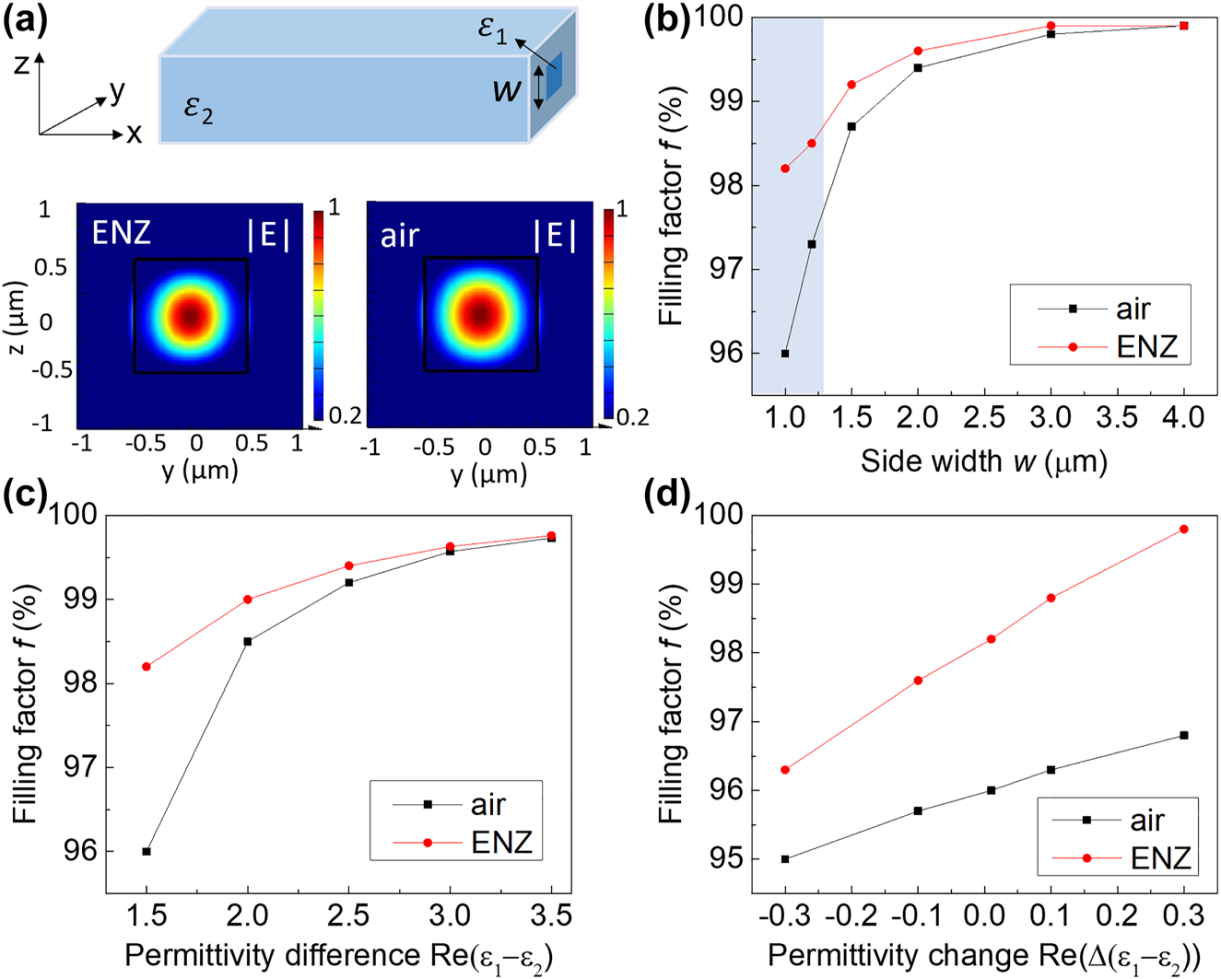
Mode confinement and the filling factor in an ENZ-based optical waveguide. (a) Scheme of a three-dimensional square waveguide with a dielectric core and the associated mode profile under transverse electric (TE) polarization. The electric field is more confined at the core regime surrounded by the ENZ environment (*ɛ*_1_ = 2.25, *ɛ*_2_ = 0.001 + 0.01*i*) compared to the air environment (*ɛ*_1_ = 2.25, *ɛ*_2_ = 1). Along the *x* axis, a smaller mode leakage via evanescent waves appears for the ENZ-based waveguide. Waveguide side width is *w* = 1 µm. (b) The mode-filling factor *f* of the ENZ-based waveguide is higher than the dielectric waveguide, especially for waveguide side lengths at the subwavelength scales (*w* < *λ* = 1.3 µm, shaded region). The permittivity is *ɛ*_1_ = 3.24 and *ɛ*_2_ = 1 for the air waveguide, and *ɛ*_1_ = 2.25 and *ɛ*_2_ = 0.001 + 0.01*i* for the ENZ-based waveguide. (c) The mode filling factor *f* of the ENZ-based waveguide is higher than the dielectric waveguide, especially when the permittivity contrast is small (Re(*ɛ*_1_ − *ɛ*_2_) < 2) between the cladding and the core region. Here *ɛ*_1_ varies and *ɛ*_2_ is fixed for both systems (*ɛ*_2_ = 0.001 + 0.01*i* and *ɛ*_2_ = 1, respectively). (d) The mode filling factor *f* is highly tolerant to variation in the real part of permittivity in the cladding layer. Here *ɛ*_1_ is fixed and *ɛ*_2_ varies for the ENZ system (*ɛ*_2_ = −0.3 + 0.01*i* to 0.3 + 0.01*i*), and vice versa for the air case (*ɛ*_1_ = 2.9 to 3.5). The plotting wavelength is *λ* = 1.3 µm for all. The side width of a square-shaped core is *w* = 1 µm.

Figure 1a depicts the scheme of a core–shell square optical waveguide. Here we used the finite-difference time-domain (FDTD) method to model the eigenmode of the optical system at the wavelength of *λ* = 1.3 µm, which is at the O band of telecommunication wavelengths [26]. Notably, ENZ materials based on semiconductor oxides exhibit nearly-zero permittivity with minor materials loss (*ɛ*_*i*_ < 0.3, where *ɛ*_*i*_ represents the imaginary part of permittivity) in the near-infrared regime, which is well suited for future CMOS integration. In contrast, metal-clad waveguides based on noble metals exhibit significant Ohmic loss at the telecommunication wavelengths, and *ɛ*_*i*_ is one order larger [28–30]. For a three-dimensional waveguide (side width *w* = 1 µm, cladding layer thickness 2.5 µm) where the core permittivity (*ɛ*_1_ = 2.25) is larger than that of the cladding layer (*ɛ*_2_ = 1), we found that the electric fields of the fundamental transverse electric (TE) mode are mostly confined within the core regime. Filling factor *f* $\left(,,=\iint_{\text{core}}\varepsilon_{i}|E{|}^{2}dx\;dy/\;\iint \varepsilon_{i}|E{|}^{2}dx\;dy\right)$ is calculated to quantify the percentage of energy density confined within the core regime; *f* increases from 90.0 to 98.2% when the cladding layer of air in the conventional waveguide is replaced by an ENZ material. Here in numerical modeling, we use *ɛ*_2_ = 0.001 + 0.01*i* to represent the ENZ materials with negligible materials loss. The waveguide side length *l* was chosen to be close to the diffraction limit (*λ*/2*n*, *n* is optical refractive index), where the fundamental TE_10_ mode can be dominantly supported. Three-dimensional optical waveguides with an ENZ cladding layer in different thicknesses also showed similar mode distributions (Figure S1). In addition, a two-dimensional slab waveguide with an ENZ cladding layer also supports fundamental optical modes at the core regime, and the filling factor is higher compared to that in the air environment (Figure S2).

Both the device size and the materials refractive index are critical in determining the optical field distribution of waveguide modes. We compare the mode confinement of ENZ and dielectric optical waveguides with different values of side widths (*w*). Interestingly, our numerical result shows that the ENZ-based waveguide sustains a higher mode-filling factor *f* than the dielectric waveguide with an air cladding layer, especially at small side widths close to the diffraction limit (Figure 1b). This behavior is attributed to the unique boundary condition at the interface of dielectric and ENZ materials, where the optical field is favorably confined in the higher-index core regime by total internal reflection. In contrast, a dielectric waveguide exhibits more radiative loss to the free space when the device size is closer to the diffraction limit.

We further studied the tolerance of mode confinement to the permittivity variation in both the cladding and the core layer. The ENZ-based waveguide can still support a highly confined optical mode under a smaller permittivity difference (*ε*_1_ − *ε*_2_ < 2) between the cladding and core regime, and the benefit over the conventional dielectric waveguide is even more prominent (Figure 1c). For the ENZ-based waveguide where the cladding layer varied slightly from *ɛ*_2_ = −0.3 to 0.3, a high filling factor *f* (>95%) is preserved, which resembles the conventional waveguide with a minor permittivity variation in *ɛ*_1_ (Figure 1d). Similar behavior is observed in a rectangular optical waveguide with varying materials permittivity (Figure S3). Such a variation in waveguide geometry can be accessible in experiments by top-down deposition and lithography processes. Notably, a high filling factor is also preserved for increased optical loss in the ENZ system from *ɛ*_2_ = 0 to *ɛ*_2_ = 0.3*i*, where *ɛ*_2_ = 0.3*i* is in the same order of the materials loss value in ENZ materials based on transparent semiconducting oxides [12].

**Figure 2:**
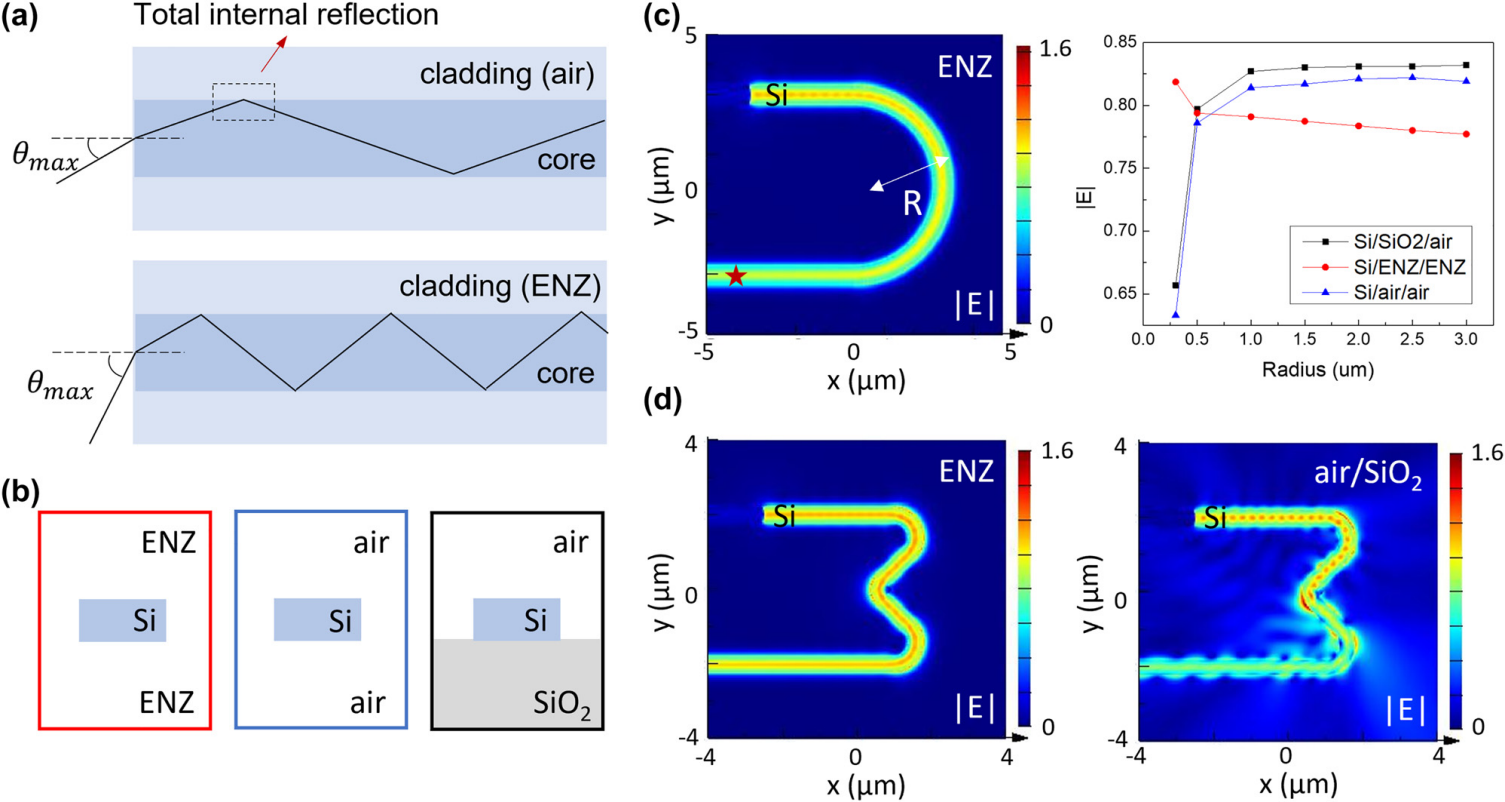
Geometry-invariant waveguides based on ENZ materials robust to waveguide bends. (a) Increased numerical aperture for waveguides with ENZ materials as the cladding layer, where *θ*_max_ can be close to 90°. (b) Cross sectional schematic of the Si waveguide within an ENZ dielectric environment, air, and on top of a glass substrate, respectively. (c) Comparison of optical transmission efficiency for Si waveguides bent to different curvatures under TE polarization. The Si waveguide within the ENZ index environment shows a higher transmission efficiency especially at small radii (*R* < 0.5 µm). A field monitor was placed in the output port (the star position). (d) Comparison of optical transmission for a bent Si waveguide in ENZ versus in air/SiO_2_ environment. Waveguide cross-section is 0.4 × 0.2 µm^2^, and the plotting wavelength is *λ* = 1.3 µm.

The permittivity contrast between the cladding and the core layers is critical in determining the numerical aperture of an optical waveguide, which defines the range of incident angles at which the light can propagate into. In principle, a lossless ENZ material as the cladding layer meets the condition of total internal reflection at any nonzero oblique angles [12, 31], which allows for a larger numerical aperture with a wider range of incident angles (Figure 2a). For example, a silica-core waveguide with an ENZ clad layer sustains a numerical aperture of 1.45, while commercially available silica fibers show a typical range between 0.05 and 0.4 [27]. Such a larger numerical aperture is favorable for waveguide bends at a smaller radius.

We compare three cases of Si waveguides where the dielectric environment is either entirely in ENZ (ENZ/Si/ENZ), in air (air/Si/air), or sitting on a silica substrate (air/Si/SiO_2_) (Figure 2b). With a small cross-section (0.4 × 0.2 µm^2^) and a submicron radius, the light can be scattered to the free space for the air/Si/air case; in contrast, light can propagate more efficiently within the curved Si waveguide if it is embedded in an ENZ environment (Figure 2c). We monitor the dependence of electric field intensity |*E*| at the output port on the waveguide radius *R*. The |*E*| intensity in the Si waveguide on SiO_2_ drops by 21% as the radius *R* decreases from 3 to 0.3 µm, which originates from the significant optical scattering loss at large curvatures (Figure S4). In contrast, the |*E*| intensity increases by 5.3% for the ENZ/Si/ENZ system, which is attributed to a lower materials loss with the reduced radius (hence a shorter propagation path) (Figure S5). Here, the optical scattering loss is no longer a limiting factor for ENZ-based waveguides especially in a submicron radius (*R* < 0.5 µm). Compared to the air/Si/SiO_2_ case, higher light propagation efficiency is achieved for the ENZ/Si/ENZ system, which can benefit low-loss and miniaturized photonic devices.

We further vary the geometry of the optical waveguides and compare the light propagation efficiency for the ENZ and dielectric systems. For a curved Si waveguide in an ENZ environment, the light propagation is robust to a 90° bend (Figure 2d). Similar behavior is observed at the telecommunication wavelength of *λ* = 1.55 µm (Figure S6). Note that a curved edge at the corner (*R* = 0.6 µm) is needed in the design to reduce back reflection of the light, and ENZ-based waveguides are robust to bends in such a small radius (Figure S7). In contrast, dielectric waveguides suffer from significant scattering loss and back reflection of the light at the curved corners, which induces lower transmission efficiency. With a star-shaped geometry defect along the path, for example, the ENZ-based waveguide is also more effective in confining the light within the core, in contrast to the strong optical scattering in the dielectric ones sensitive to the waveguide geometry (Figure S8). Besides TE modes, we found that transverse magnetic (TM) modes can be supported in ENZ-based waveguides as determined by input light polarization and height of the waveguide cross section and the light propagation is robust to bends at large curvatures (Figure S9).

Compared to traditional waveguides based on dielectrics, we found that the crosstalk between ENZ-based waveguides is significantly reduced at a subwavelength separation. Two adjacent dielectric waveguides show crosstalk in between with a small separation of 50 nm, while the ENZ-based waveguides can maintain the light propagation in the original input channel (Figure S10). The reduced crosstalk can be explained by a smaller penetration depth of the evanescent wave [32] at the dielectric/ENZ interface, which reduces the coupling coefficient between optical waveguides. Such a system based on ENZ materials can reduce the coupling between waveguides at submicron separations and be beneficial for integrated, ultracompact photonic circuits [33]. Notably, the ENZ-based waveguide can split part of the light beam to another channel as they cross, which shows the promise to function as a beam splitter at submicron scales at designated wavelength regimes without needing a bulky dichroic prism [32] (Figure S11).

At small device sizes close to the diffraction limit, the fundamental TE_10_ mode is effectively confined within the waveguide even when the materials loss of semiconducting oxides is included (*ɛ*_*i*_ = 0.3) (Figure S12a). The radius of bends is restricted by the device geometry and can be as small as close to half the waveguide side length *l* (Figure S12b). Importantly, the ENZ layer can clad at only the critical bending and crossing regions of the dielectric waveguides, where the optical loss is reduced even when the materials loss is present (*ɛ*_*i*_ = 0.3, Figure S13). For example, higher light transmission is achieved when an ENZ matrix (1 × 1 µm^2^) is placed in the 90° bending region of a Si waveguide. The materials loss in the ENZ system can be minimized by using photonic crystals made of dielectrics for effectively-zero permittivity [34, 35] or by operating at the cutoff frequency of TE_10_ mode in optical waveguides [36]. In addition, the materials loss in semiconducting oxides can be compensated by dopants such as Er^3+^ ions as the optical gain through ion implantation [37], which offers prospects for future studies.

**Figure 3:**
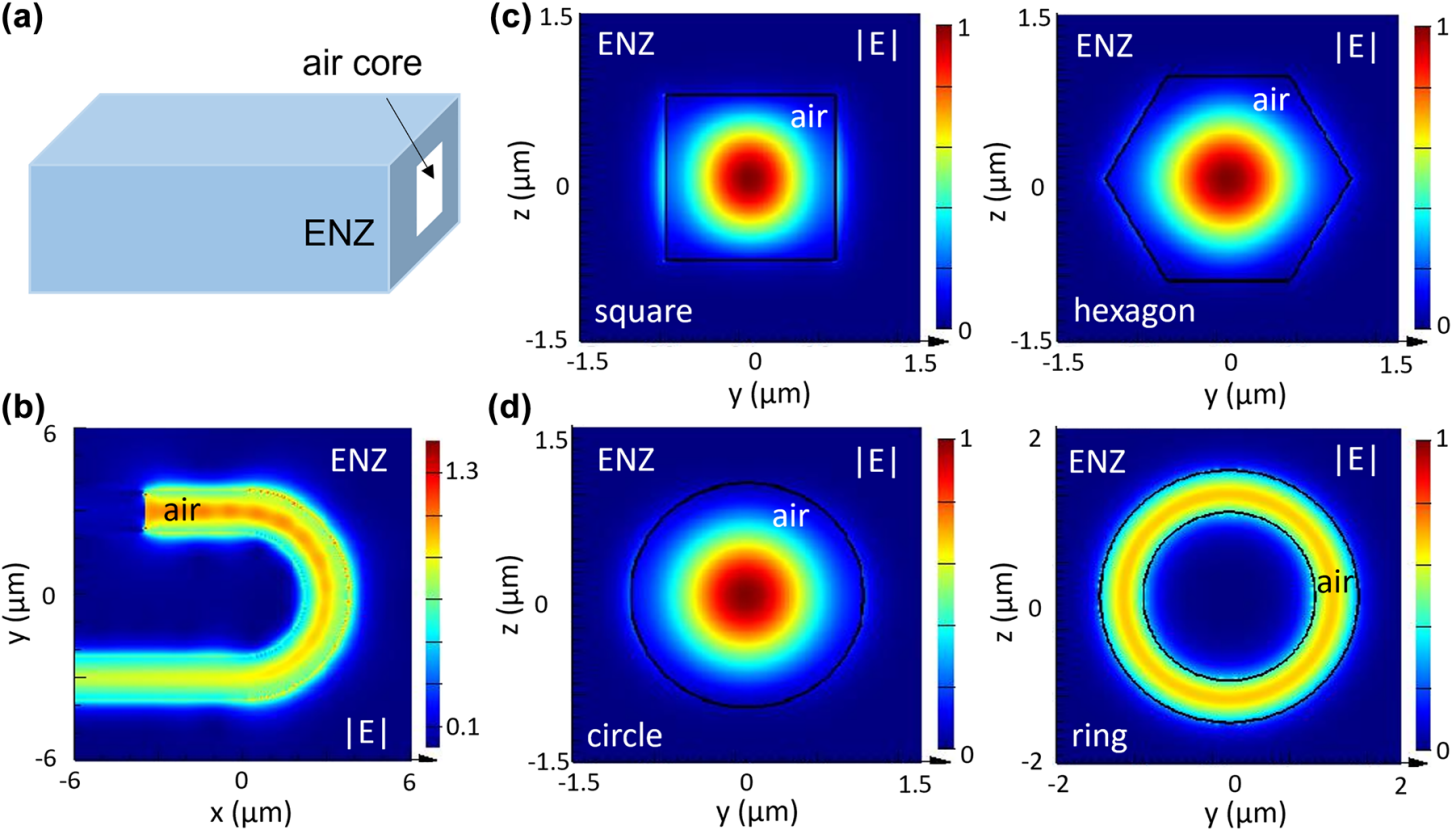
Light propagation within air-core optical waveguides based on ENZ materials. (a) Scheme of a three-dimensional layout. (b) The light propagation with a curved air-core waveguide (side length *w* = 1.5 µm, radius *R* = 3 µm) embedded in the ENZ material under TE polarization. (c) Mode confinement in a zero-index environment with a square or a hexagonal waveguide cross section. (d) Mode confinement in a zero-index environment with a circular or a ring-shaped cross section. The mode profiles were plotted under TE polarization. The plotting wavelength is *λ* = 1.3 µm.

Besides the higher mode confinement enabled by the ENZ cladding layer, we investigate a new waveguide design that can potentially reduce optical loss based on a hollow structure. Figure 3a depicts an air-core waveguide where an ENZ material serves as the cladding layer. From FDTD modeling, the large index contrast between ENZ and air at the boundary supports confined optical modes within the air core, and light propagates efficiently within this hollow waveguide (Figure 3b). Such a design can minimize the materials absorption at the core regime to realize ultralow-loss optical waveguides, and the incorporation of gas or liquid flow with the air core could benefit optical monitoring and sensing. It could also facilitate light propagation within on-chip optical waveguides over a broader wavelength range beyond the telecommunication bands [32]. Besides a square-shaped waveguide, we found that the fundamental TE mode is also supported in optical waveguides with a hexagonal, circular, or ring-shaped cross section (Figure 3c and d). Such a sustained mode distribution invariant to the change in cross-section geometries is also observed at the telecommunication wavelength of *λ* = 1.55 µm (Figure S14). We further changed the geometry of the air-core waveguide by increasing the cross-section size or misaligning the waveguide core axis, and found that light propagation is very robust to local geometry variation (Figure S15).

## 3 Conclusions

In summary, we demonstrate that using ENZ materials as the cladding layer in optical waveguides introduces significant electric field confinement with the benefit of reduced optical loss, and the light propagation is robust to geometry variations at the boundaries. The lower optical loss originates from stronger electric field confinement for device sizes close to the diffraction limit and reduced crosstalk between waveguides at a subwavelength separation. Minimized materials absorption can be accessed in a hollow waveguide because of the air core. Our ENZ-based waveguides show invariance to geometry change such as local defects and bends at large curvatures, which offers critical insights into the future miniaturized and low-loss photonic integrated circuits. In addition, the strongly confined optical fields within the air core in the hollow waveguide may find applications in optical sensing, environmental monitoring, and medical diagnosis.

## 4 Methods

### 4.1 Electromagnetic modeling

Finite-difference time-domain (FDTD) calculations with commercial software (FDTD Solutions, Mode Solutions, Lumerical Inc., Vancouver, Canada) were used to model the eigenmode field distribution and light propagation of optical waveguides based on near-zero-index materials. We used a uniform mesh size (<10 nm) to ensure the accuracy of electric and magnetic field calculations within the near-zero-index region. The eigenmode analysis and electric field distribution plots are extracted from *E*_x_, *E*_y_, and *E*_z_ values at different grid sites in the FDTD simulations.

## Supporting information

The online version of this article offers Supplementary Material.

## Supplementary Material

Supplementary Material Details
