# Supplementary material for: Low-loss, geometry-invariant optical waveguides with near-zero-index materials
Source: Nanophotonics. 2022 Nov 7;11(21):4747–53. doi: 10.1515/nanoph-2022-0445 (PMC11501372; doi:10.1515/nanoph-2022-0445)
Supplement: Supplementary file 1 — Supplementary Material Details [file j_nanoph-2022-0445_suppl.pdf]

Low-loss, geometry-invariant optical waveguides with near-zero-index materials

Danqing Wang[1,2]*, Kaichen Dong[1], Jingang Li[3], Costas Grigoropoulos[3], Jie Yao[1], Jin Hong[4], Junqiao Wu[1]*

[1]Department of Materials Science and Engineering, University of California, Berkeley, Berkeley, California, 94720, USA

[2]Miller Institute, University of California, Berkeley, Berkeley, California, 94720, USA

[3]Department of Mechanical Engineering, University of California, Berkeley, Berkeley, California, 94720, USA

[4]To be updated

*Corresponding author: danqingwang2018@u.northwestern.edu; wuj@berkeley.edu

Supplementary Fig. S1. Maintained mode confinement for a finite-sized epsilon-near-zero (ENZ) cladding layer S2

Supplementary Fig. S2. Two-dimensional optical waveguide modes with ENZ materials ..S3

Supplementary Fig. S3. Mode confinement tolerant to the minor permittivity change in a rectangular waveguide S4

Supplementary Fig. S4. Light propagation in curved Si waveguides....................................S5

Supplementary Fig. S5. Light propagation with different ENZ materials loss S6

Supplementary Fig. S6. Geometry-invariant ENZ waveguides at 1.55 µm S7

Supplementary Fig. S7. ENZ-based waveguides with bends at small curvatures S8

Supplementary Fig. S8. ENZ-based waveguides with geometry defects..............................S9

Supplementary Fig. S9. Transverse magnetic modes in ENZ-based waveguides...............S10

Supplementary Fig. S10. Cross coupling in dielectric and ENZ-based waveguides...........S11

Supplementary Fig. S11. ENZ-based waveguides with crossings.......................................S12

Supplementary Fig. S12. Mode distribution with materials loss..S13

Supplementary Fig. S13. Light propagation with materials loss..S14

Supplementary Fig. S14. Mode profiles in air-core ENZ-based waveguides at 1.55 µm....S15

Supplementary Fig. S15. Air-core ENZ-based waveguides with geometry variation S16

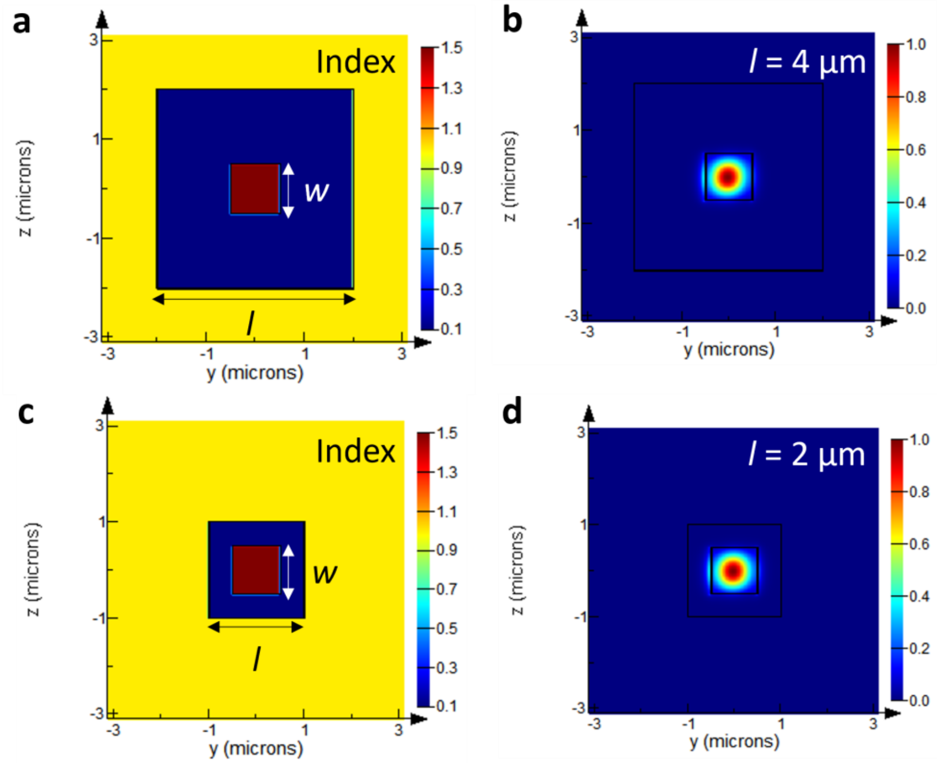

Figure S1. The mode confinement is maintained with a finite-sized ENZ cladding layer. (**a**) Refractive index plot for a finite-sized waveguide with core width w = 1 µm and cladding layer width l = 4 µm. (**b**) The mode distribution plot where the mode filing factor f resembles the case with an infinite-thick ENZ cladding layer. (**c**) Refractive index plot for a finite-sized waveguide with core width w = 1 µm and cladding layer width l = 2 µm. (**d**) The mode distribution plot where the mode filing factor f resembles the case with an infinite-thick ENZ cladding layer. The plotting wavelength is λ = 1.3 µm.

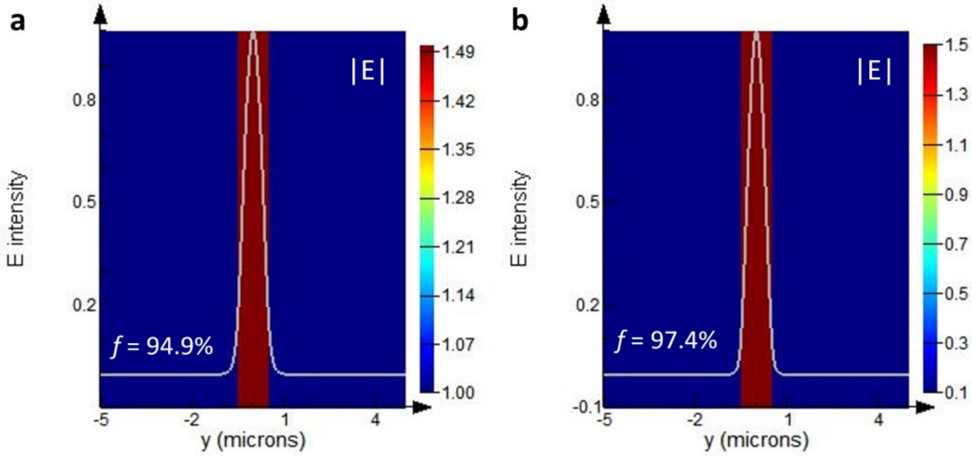

Figure S2. 2D optical waveguides with ENZ materials. (**a**) Mode confinement in near-zero-index environment with a core at permittivity ε_1 = 2.25 and a cladding material at ε_2 = 1. (**b**) Mode confinement in near-zero-index environment with a core at permittivity ε_1 = 2.25 and a cladding material at ε_2 = 0.01. The waveguide side width is w = 1 μm, and the plotting wavelength is λ = 1.3 μm.

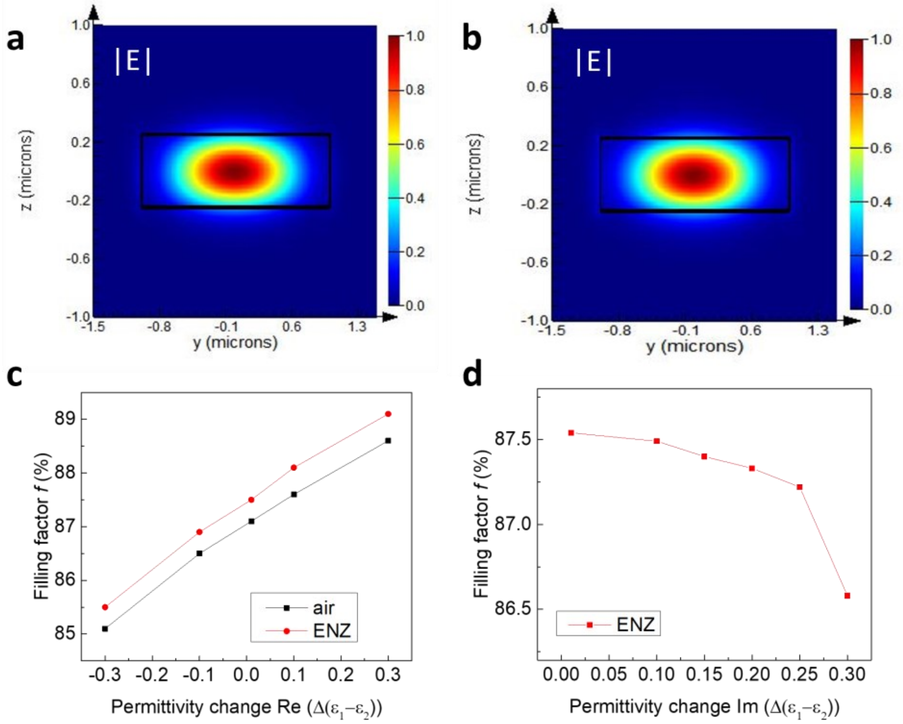

Figure S3. The mode confinement is tolerant to the minor permittivity changes in a rectangular waveguide. (**a**) Mode confinement in near-zero-index environment with a rectangular core at $\varepsilon_1 = 0.1$, and (**b**) ε_1 = -0.1. The filling factor is f = 88.2% and f = 87.0%, respectively. The waveguide cross section is 2×0.5 μm^{2,} and the plotting wavelength is 1.3 μm. (**c**) The mode filling factor is tolerant to the change of the real part of permittivity in the cladding layer. (**d**) The mode filling factor is tolerant to the change of the imaginary part of permittivity in the cladding layer ($\Delta\varepsilon < 0.3$). Note that $\varepsilon_2 = 0.3i$ represents the loss of ENZ materials based on transparent semiconducting oxides.

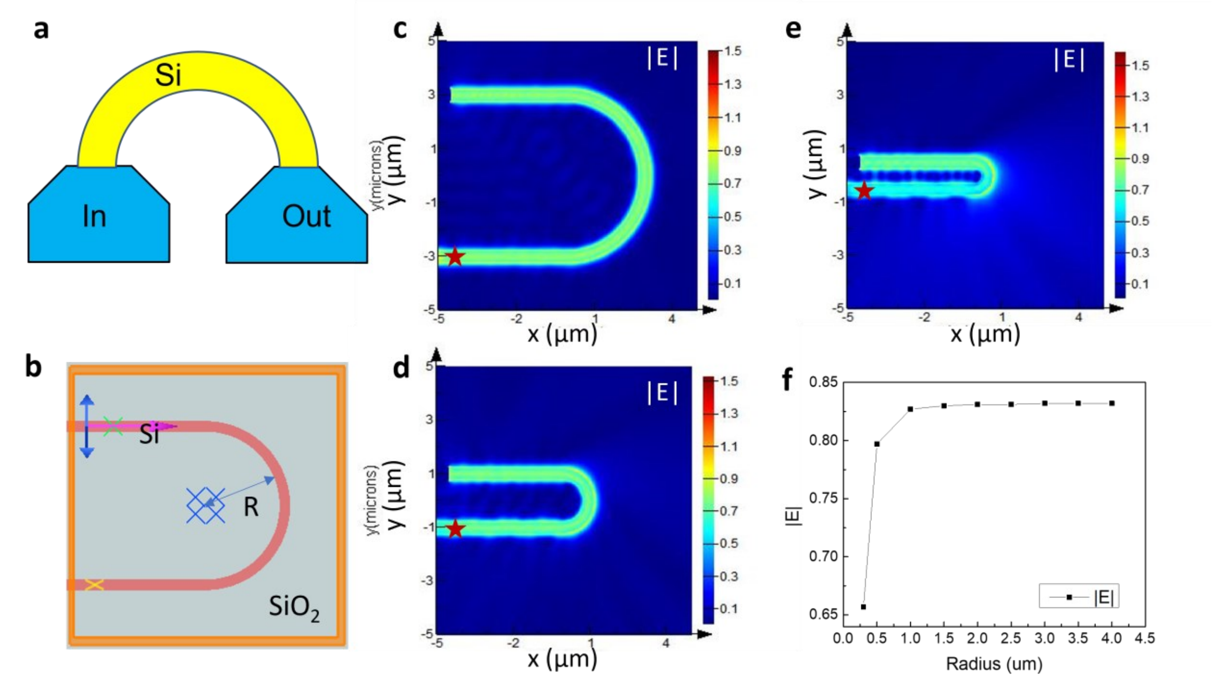

Figure S4. Light propagation in curved Si waveguides. (**a**) Scheme of a curved Si waveguide. (**b**) The modeling setup in finite-difference time-domain (FDTD) simulations for a Si waveguide on a SiO_2 substrate at different radius R. The light propagation within Si waveguide at a radius of (**c**) R = 3 μm (**d**) R = 1 μm, and (**e**) R = 0.5 μm. (**f**) The electric field intensity at the output port side as a function of the waveguide radius R. Waveguide side length is 0.4 μm, and the plotting wavelength is λ = 1.3 μm. The waveguide side length was chosen to be close to the diffraction limit (λ /2n), where the fundamental TE_{10} mode can be dominantly supported. For example, for a Si-core waveguide with a side length l = 0.4 μm, the cut-off operation wavelength of TE_{10} mode is at 2.8 μm, and the cut-off operation wavelength of TE_{20} mode is at 1.4 μm. Higher-order modes are not able to be excited in the system, which benefits the transfer of a well-defined signal in integrated circuits.

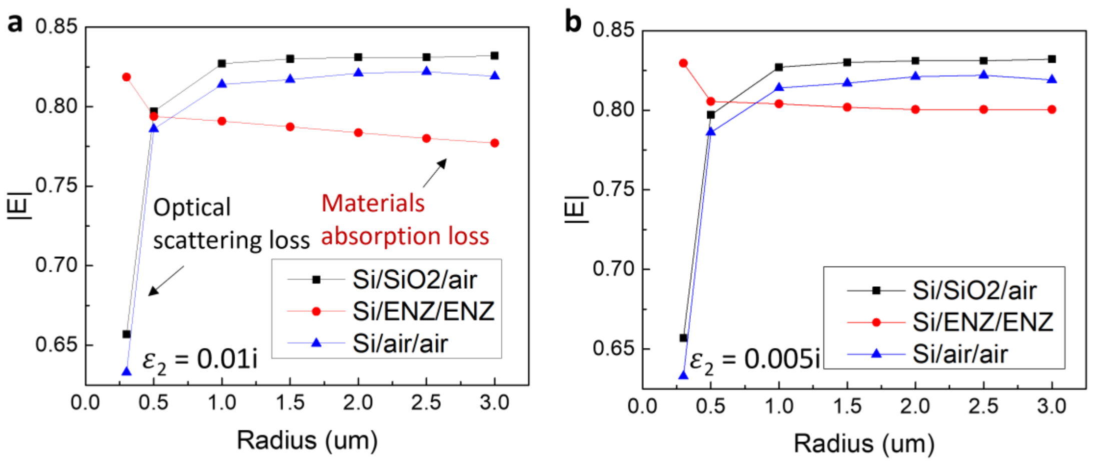

Figure S5. Light propagation at different ENZ materials loss. The |E| field intensity at the output port for a Si waveguide at different curvatures with (**a**) ENZ permittivity $\varepsilon_2 = 0.01i$, and (**b**) ENZ permittivity $\varepsilon_2 = 0.005i$. The plotting wavelength is $\lambda = 1.3$ µm. In numerical modeling, a lower materials loss can be achieved by decreasing the imaginary part of permittivity of the ENZ materials, which led to a higher electric field intensity. Hence a Si waveguide with a smaller radius that exhibits a shorter light path can maintain a higher light propagation efficiency over distance.

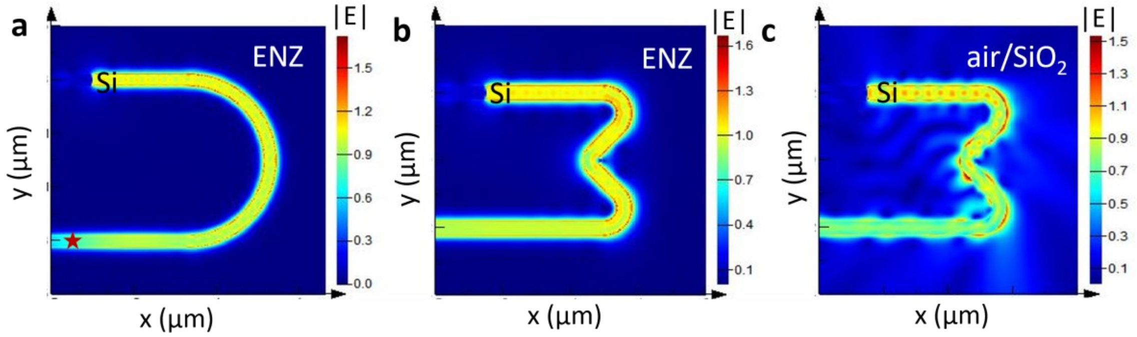

Figure S6. Geometry-invariant ENZ waveguides at the telecommunication wavelength of 1.55 µm. (a) The light propagation within Si waveguide at radius R = 3 µm under transverse electric (TE) polarization. (**b-c**) The comparison of optical transmission efficiency for a bent Si waveguide in ENZ or air/SiO_2 environment. The waveguide side length is w = 0.4 µm.

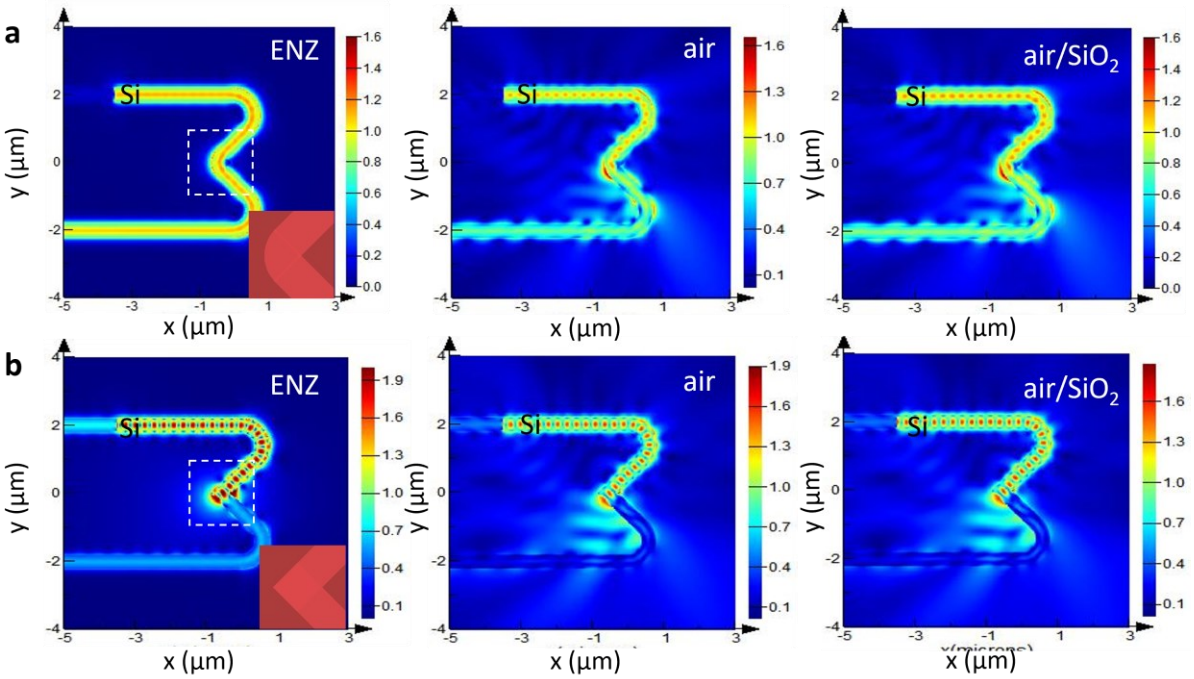

Figure S7. The comparison of light propagation in bent Si waveguides. (**a**) Light propagation in a curved Si waveguide with a curved 90° bend. (**b**) Light propagation in a curved Si waveguide with a straight, sharp 90° bend. Compared to the dielectric waveguides, much reduced optical scattering and less back reflection of the light at the curved corners is observed in ENZ-based optical waveguides. The plotting wavelength is $\lambda = 1.3$ μm.

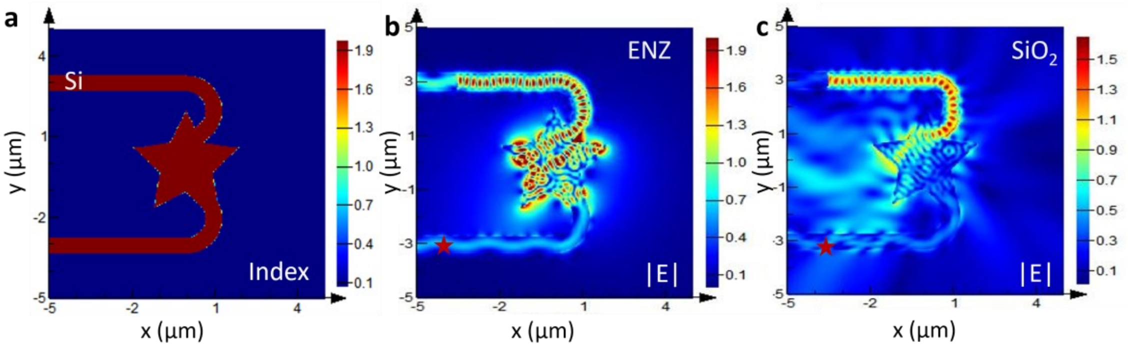

Figure S8. The comparison of light propagation in an ENZ waveguide with local defects. (**a**) The refractive index distribution plot in FDTD modeling. (**b**) Light propagation in a Si waveguide with a local star-shaped defect in an ENZ environment and, (**c**) on a SiO_2 substrate. The plotting wavelength is λ = 1.3 μm. The electric field intensity |E| at the output port is 0.61 and 0.39, respectively. Compared to the dielectric waveguide where the light scatters to the free space at irregular boundary corner, the ENZ-based waveguide can effectively confine the light within the waveguide core regime.

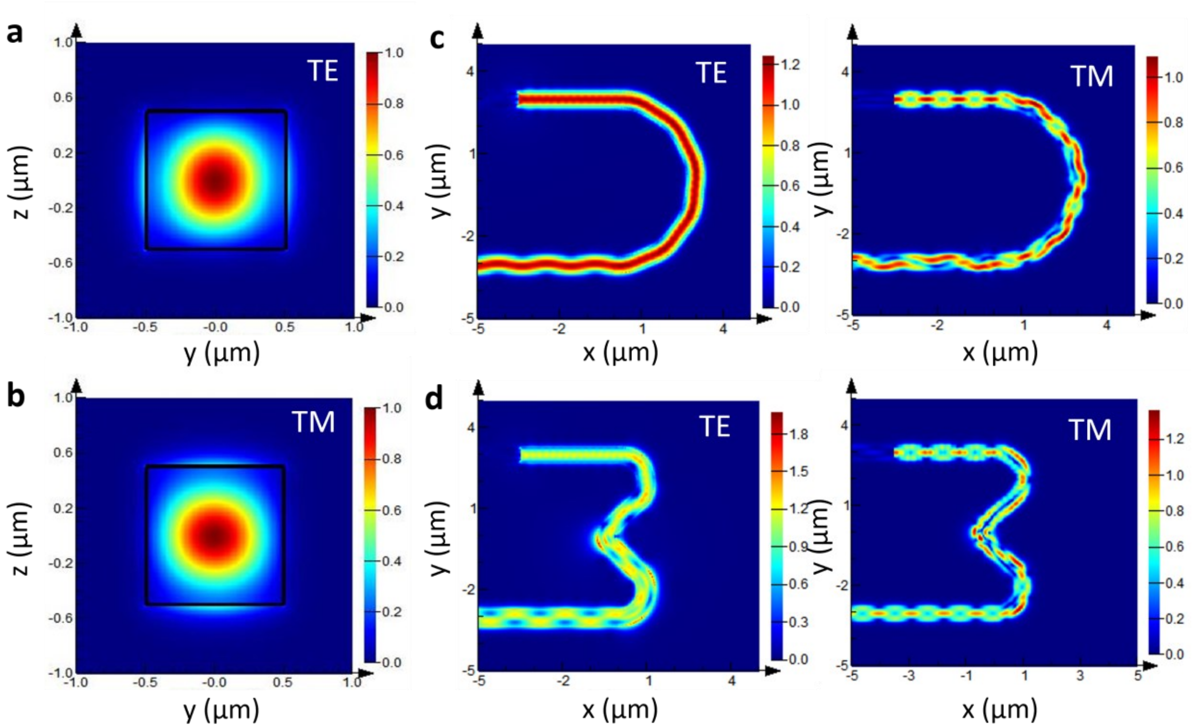

Figure S9. Transverse magnetic (TM) modes supported in ENZ-based waveguides. (**a-b**) The field distribution of TE and TM modes in a Si waveguide with ENZ materials as the cladding layer. (**c**) Light propagation with a curved ENZ-based waveguide (radius R = 3 μm) under TE and TM polarization. The side length is l = 0.4 μm and height is h = 0.4 μm for TM polarization. (**d**) Light propagation with a bent ENZ-based waveguide under TE and TM polarization. The plotting wavelength is λ = 1.3 μm.

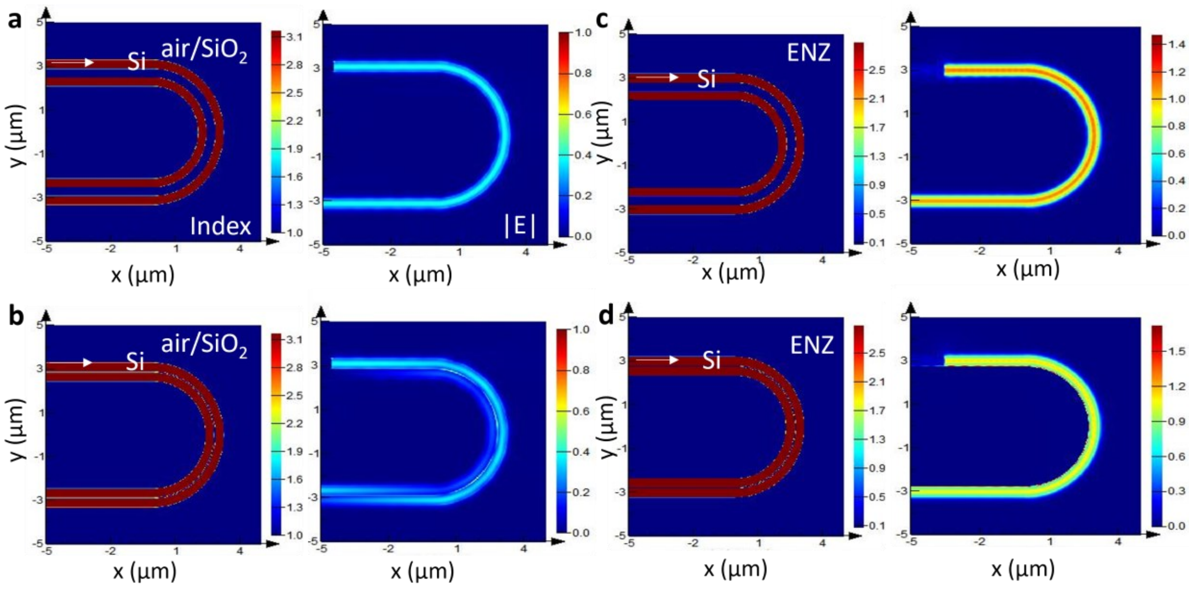

Figure S10. Comparison of cross coupling in dielectric waveguides and ENZ-based waveguides. The index distribution and electric field distribution plots for two adjacent Si waveguides on a SiO_2 substrate with a separation of (**a**) 0.4 µm and, (**b**) 0.05 µm. The index distribution and electric field distribution plots for two adjacent Si waveguides within ENZ environment with a separation of (**c**) 0.4 µm and, (**d**) 0.05 µm. Compared to traditional waveguides based on dielectrics, the crosstalk between ENZ-based waveguides is significantly reduced at a subwavelength separation. The plotting wavelength is λ = 1.3 µm.

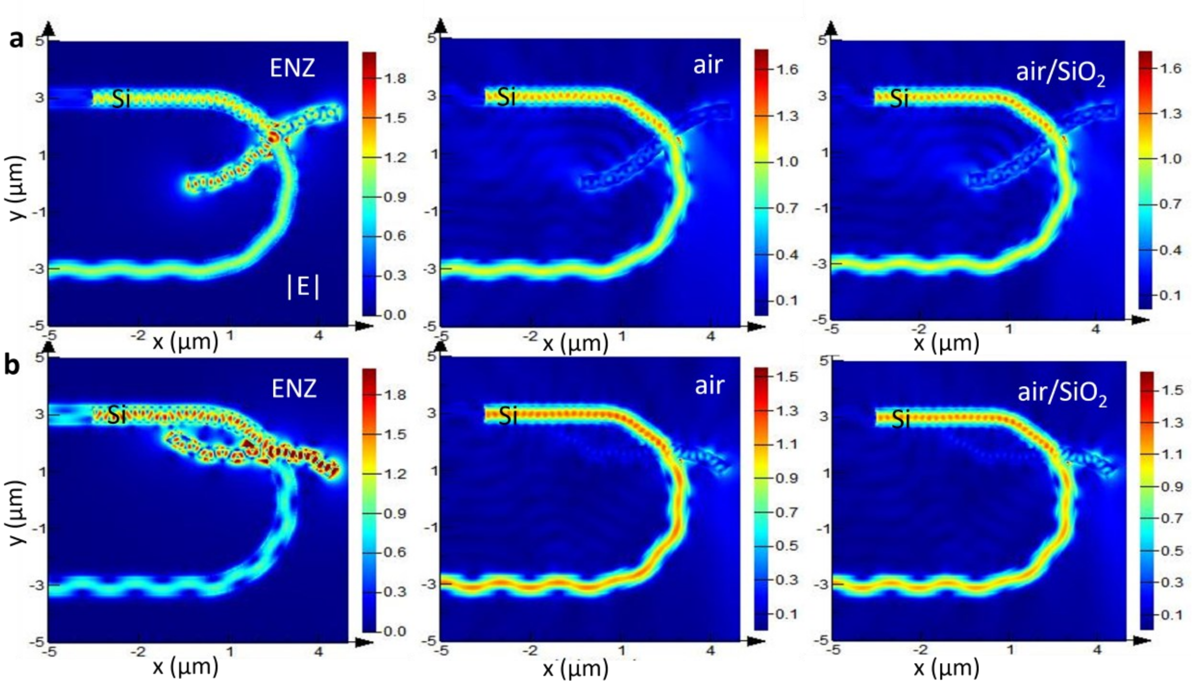

Figure S11. The comparison of light propagation in ENZ waveguides with crossings. (**a**) Light propagation in a Si waveguide (R = 3 μm) with crossing in the center in ENZ environment, air environment, and on a SiO_2 substrate. (**b**) Light propagation in a curved Si waveguide with crossing at the corner. The plotting wavelength is λ = 1.3 μm. The ENZ waveguide can split part of the light beam to another channel as they cross with each other, which shows the promise to function as a beam splitter at submicron scales at designated wavelength regimes without needing a dichroic prism. To be compared, the dielectric waveguide is more robust to these crossings, and light is preserved within the initial waveguide channel where the light was injected.

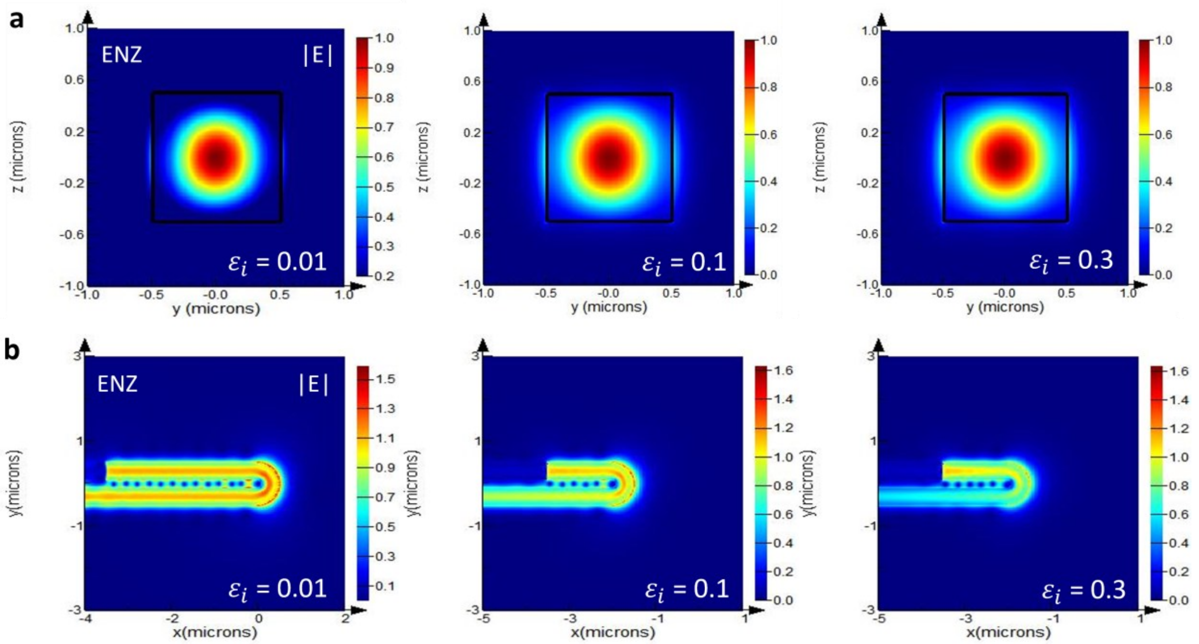

Figure S12. The mode distribution in ENZ-based waveguides with materials loss. (**a**) The field distribution of the fundamental TE_{10} mode in a SiO_2 waveguide with ENZ materials as the cladding layer at different materials losses. The waveguide side length is $l = 1$ μm. (**b**) Light propagation with a curved ENZ-based Si waveguide (radius R = 0.3 μm) under TE polarization. The waveguide cross section is 0.4×0.2 μm^2.

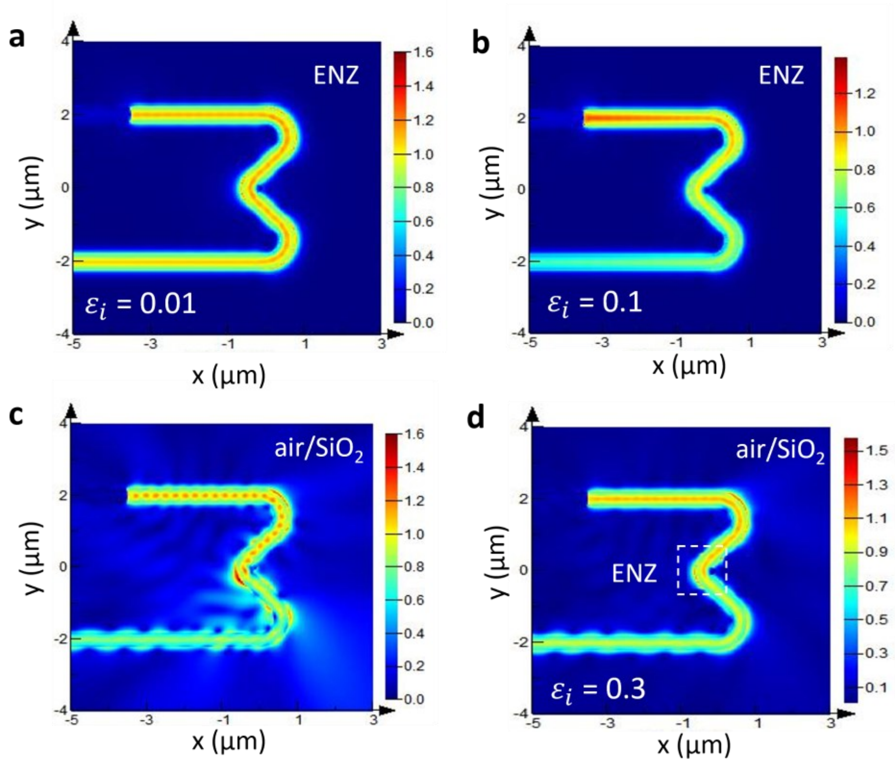

Figure S13. Light propagation in ENZ-based waveguides with materials loss. The light propagation in a Si waveguide with a cladding ENZ layer at (**a**) ε_i = 0.01 and, (**b**) ε_i = 0.1. (**c**) The light propagation in a bent Si waveguide with a SiO_2 substrate and air as the superstrate. The electric field intensity |E| at the output port is 0.81. (**d**) The field plot when an ENZ matrix (1×1 μm^2) is placed in the 90° bending region. A materials loss of ε_i = 0.3 was included, which matches the state-of-art value of transparent semiconducting oxides at 1.3 μm. The electric field intensity |E| at the output port is 0.9. The pump light is under TE polarization.

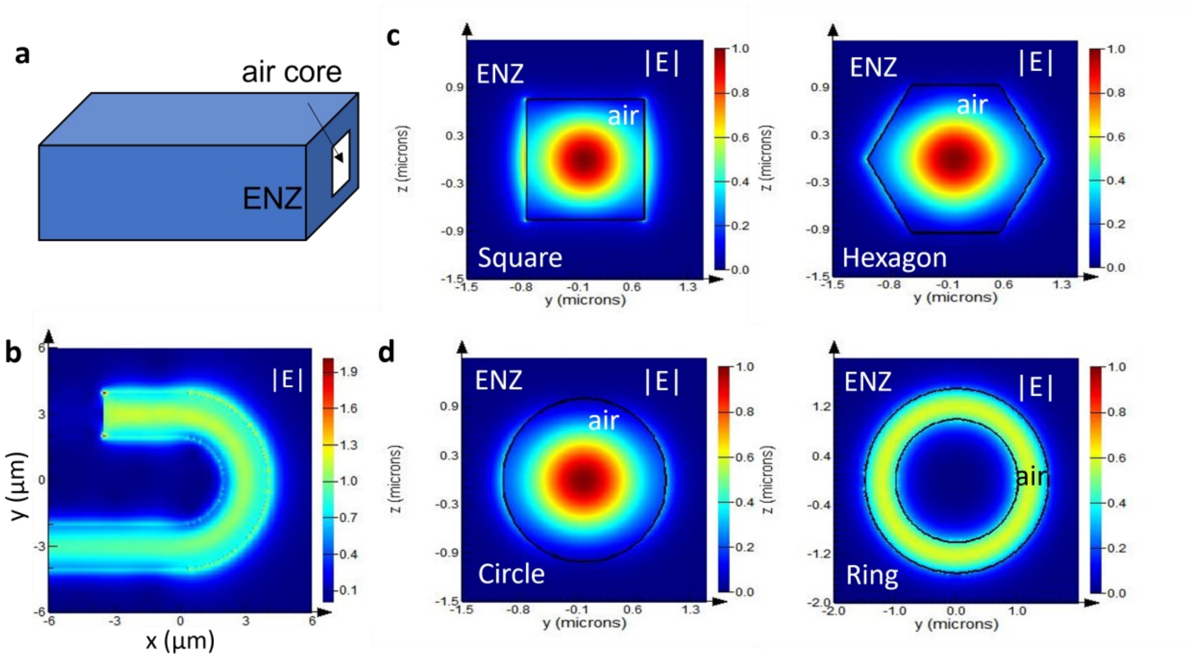

Figure S14. Air-core ENZ waveguides with the operation wavelength at 1.55 μm. (**a**) Scheme of a 3D layout. (**b**) The light propagation with an air-core waveguide (side length w = 2 μm) with the ENZ embedding layer under TE polarization. (**c**) Mode confinement in near-zero-index environment with a square and a hexagon waveguide cross section. (**d**) Mode confinement in near-zero-index environment with a circle and a ring-shaped cross section. The waveguide side length is 1.5 μm for the square and 1 μm for the hexagon. The radius is 1 μm for the circle, and 1 μm (inner) and 1.5 μm (outside) for the ring. The plotting wavelength is λ = 1.55 μm.

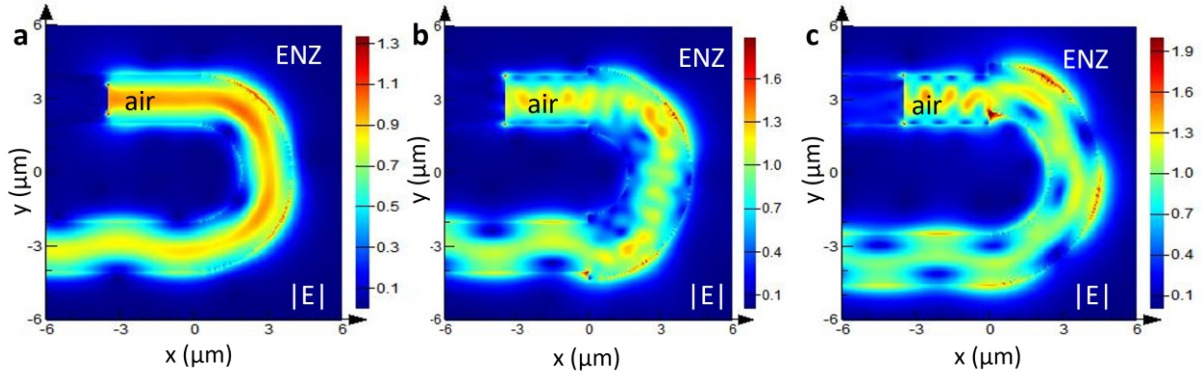

Figure S15. Air-core ENZ waveguides with geometry deformation. The light propagation in an air-core waveguide with (**a**) a radius R = 3 μm and a cross-section width w = 2 μm and, (**b**) a larger cross section width (w = 2.6 μm) in the middle and, (**c**) mis-aligned waveguide cross section. The pump light is under TE polarization, and the plotting wavelength is λ = 1.3 μm.
